# Pediatric Use of Off‐Label Medicines and Nonevidence‐Based Supplements for COVID‐19: Findings From the 2015 Pelotas Birth Cohort Study (Brazil)

**DOI:** 10.1002/pds.70460

**Published:** 2026-08-23

**Authors:** Andréa Dâmaso Bertoldi, Fernando Silva Guimarães, Alexandra Crispim Boing, Marysabel Pinto Telis Silveira, Pedro Curi Hallal, Mariângela Freitas da Silveira

**Affiliations:** ^1^ Postgraduate Program in Epidemiology Federal University of Pelotas Pelotas Brazil; ^2^ Federal University of Santa Catarina Florianópolis Brazil; ^3^ Multicenter Postgraduate Program in Physiological Sciences Federal University of Pelotas Pelotas Brazil; ^4^ University of Illinois Urbana‐Champaign Champaign Illinois USA

**Keywords:** cohort studies, COVID‐19, off‐label drug use, pediatrics

## Abstract

**Purpose:**

Pediatric off‐label medicine use increased during the COVID‐19 pandemic, exposing children to nonevidence‐based treatments. We described the use of nonevidence‐based COVID‐19 medicines and supplements among children from the 2015 Pelotas Birth Cohort, according to sociodemographic and health‐related characteristics.

**Methods:**

Data from the 6–7‐year follow‐up were analyzed. Mothers/caregivers reported children's use of nonevidence‐based medicines (azithromycin, chloroquine/hydroxychloroquine, ivermectin, nitazoxanide) and supplements (vitamin C, vitamin D, and zinc) along with sociodemographic and health‐related characteristics. Two scores were analyzed: (1) child's COVID‐19 burden score (considering negative experiences such as death or separation from a close person due to COVID‐19, and risk factors such as living with older adults or having asthma, bronchitis, or obesity) and (2) preventive behavior score (limited outdoor exposure during COVID‐19 and vaccine uptake against COVID‐19). Hierarchical logistic regression analyses were used to identify factors associated with nonevidence‐based medicine and supplement use.

**Results:**

Among 3098 children interviewed after COVID‐19 vaccines became available in the municipality, 13.9% (95% CI 12.7–15.2) used nonevidence‐based medicines and supplements for COVID‐19. Overall, the use of nonevidence‐based medicines and supplements increased with higher COVID‐19 burden scores and decreased with higher preventive behavior scores. Unvaccinated children had 2.57‐fold higher odds of using off‐label medicines than vaccinated children.

**Conclusions:**

These findings highlight behavioral responses during a health emergency and suggest that public health policies should strengthen evidence‐based risk communication and discourage inappropriate medicine use during future health emergencies.

## Key Points

- During the pandemic, health misinformation in Brazil led to the promotion of nonevidence‐based medicines and supplements for COVID‐19.
- Among 3098 children from a Brazilian cohort, 13.9% used nonevidence‐based medicines and supplements for COVID‐19.
- Ivermectin and azithromycin were the most frequently used off‐label medicines, while vitamins C and D were the most frequently used nonevidence‐based supplements among children.
- Families with negative experiences (death or separation from a relative due to COVID‐19), comorbidities, or older cohabitants were more likely to adopt nonevidence‐based treatments. Conversely, adherence to WHO recommendations (vaccination and reduced outdoor exposure) was associated with reduced use.

## Plain Language Summary

Off‐label medicine use refers to prescribing drugs for purposes not approved by a country's regulatory authority. In Brazil, during COVID‐19, the off‐label use of medicines and nonevidence‐based supplements increased, largely driven by health misinformation. This study aimed to describe the use of nonevidence‐based medicines and supplements for COVID‐19 among children from the 2015 Pelotas Birth Cohort. We analyzed data from the 6–7‐year follow‐up, and mothers were asked about the use of the following medicines and supplements to treat or prevent COVID‐19: azithromycin, chloroquine/hydroxychloroquine, ivermectin, nitazoxanide, vitamin C, vitamin D, and zinc. Among 3098 children, we found that 13.9% (95% CI 12.7–15.2) used nonevidence‐based medicines and supplements for COVID‐19. Ivermectin and azithromycin were the most frequently used off‐label medicines, while vitamins C and D were the most frequently used nonevidence‐based supplements. Children with negative experiences (death or separation from a relative due to COVID‐19) and COVID‐19 risk factors (living with older adults or having asthma, bronchitis, or obesity) during the pandemic were more likely to use nonevidence‐based medicines and supplements compared to children who adopted preventive behaviors (vaccination and reduced outdoor exposure). These findings may inform strategies to promote safer practices for children during health emergencies.

## 1 Introduction

The COVID‐19 pandemic marked a turning point for health misinformation (unreliable, shared information) and disinformation (deliberately misleading information) [1, 2], prompting the World Health Organization (WHO) to address the global impact and management of infodemics at the 1st Infodemiology Conference in 2020 [3]. In Brazil, health misinformation promoted the use of off‐label medicines for COVID‐19 [4] and the rejection of WHO‐recommended preventive measures, including mask‐wearing, social distancing, and vaccination [4, 5].

By January 2021, Brazil had the second‐highest number of COVID‐19 deaths, and an estimated 156 582 deaths could have been avoided if the country had matched the global average COVID‐19 response [6]. This context worsened health inequalities during the pandemic. Data collected between May and June 2020 from EPICOVID‐19, a large population‐based serological survey of SARS‐CoV‐2 antibodies, showed that one in four individuals either did not seek healthcare despite a health problem or missed a routine medical examination during the early months of the pandemic (March 2020) [7].

Patterns of medicine use changed during COVID‐19. The therapeutic and prophylactic use of nonevidence‐based medicines (azithromycin, hydroxychloroquine, ivermectin, and nitazoxanide) and supplements (vitamin C, vitamin D, and zinc) increased, driven by the Brazilian government, which promoted the “COVID kit” [4, 8]. Supported by the medical organization Physicians for Life, the “COVID kit” promoted “early treatment” with nonevidence‐based medicines and supplements [4]. An analysis of Brazilian Health Regulatory Agency (Anvisa) sales and adverse drug reaction (ADR) data showed that indiscriminate use of these medicines correlated with higher ADR rates [8].

The rise in use of these medicines prompted Brazil to revise dispensing regulations. In March 2020 (Anvisa RDC No. 351/2020), chloroquine, hydroxychloroquine, and nitazoxanide were classified as specially controlled drugs [9] to curb misuse and preserve supplies. In July 2020 (Anvisa RDC No. 405/2020), ivermectin was placed under temporary exceptional control [10]. This measure lasted only 30 days (Anvisa RDC No. 420/2020), after which ivermectin and nitazoxanide returned to their previous dispensing status [11]. Hydroxychloroquine, however, remained under special control [11]. Azithromycin already required prescription retention, and its dispensing status remained unchanged. Likewise, supplements and vitamins remained over‐the‐counter (OTC) products and did not require a prescription.

As in adults, pediatric COVID‐19 treatment relied on off‐label use, driven by potential treatment benefits and the absence of standard treatment [9, 12]. Children were also considered to have milder COVID‐19 than adults [13]. During the pandemic, children and infants were exposed to off‐label medicines, as their use often depended on parents' or caregivers' perceptions [14, 15]. Our primary objective was to describe the period prevalence of off‐label use of medicines (azithromycin, chloroquine/hydroxychloroquine, ivermectin, nitazoxanide) and nonevidence‐based supplements (vitamin C, vitamin D, and zinc) for COVID‐19 among children aged 6–7 years in the 2015 Pelotas Birth Cohort Study, during 2021–2022, according to treatment or preventive use. The secondary objective was to investigate predictors of the use of these medicines and supplements across sociodemographic, health, and pandemic‐related factors using an a priori‐defined analytical framework.

## 2 Methods

### 2.1 Study Design and Population

We analyzed data from the 2015 Pelotas Birth Cohort Study, which followed all live births in Pelotas, Brazil, in 2015. At baseline, the cohort sample comprised 4275 participants assessed. More details are available elsewhere [16, 17]. We used data from the 6–7‐year follow‐up, conducted between November 2021 and December 2022. At 6–7 years, 3867 participants were assessed (follow‐up rate: 92.0%). The analytical sample included 3098 participants, regardless of their COVID‐19 status, who were assessed after COVID‐19 vaccination became available through the Pelotas Public Health System (January 19, 2022) and had valid information on the use of nonevidence‐based medicines and supplements (Figure S1). All COVID‐19 prevention and control protocols were followed during the study.

All cohort assessments from birth to 6–7 years were approved by the Research Ethics Committee of the Federal University of Pelotas (at 6–7 years follow‐up: 51789921.1.0000.5317). Mothers and caregivers provided written informed consent.

### 2.2 Outcomes

In the 6–7‐year follow‐up study, we asked mothers and caregivers about their child's use of medicines and supplements for COVID‐19 treatment or prevention from the start of the pandemic until the interview using the question: “Did <Child> use any of these medicines during the pandemic to prevent or treat COVID‐19?” Subsequently, the use of the following medicines and supplements was collected (yes/no): azithromycin, chloroquine/hydroxychloroquine, ivermectin, nitazoxanide, vitamin C, vitamin D, and zinc. For each positive answer, we asked whether it was prescribed by a physician (yes/no) and its indication (prophylactic, therapeutic, or both) (Table S1). We estimated the prevalence of use for all medicines and supplements, and separately for medicines only (azithromycin, chloroquine/hydroxychloroquine, ivermectin, and nitazoxanide) and supplements only (vitamin C, vitamin D, and zinc). The use of any investigated medicine and supplement was defined as nonevidence‐based therapy, since there was no standard treatment for COVID‐19 in Brazil during the study period. Throughout the manuscript, medicine use is referred to as off‐label, whereas supplement use is referred to as nonevidence‐based. When both are mentioned together, the term “nonevidence‐based medicines and supplements” is used.

### 2.3 Covariates

We included the following covariates, collected through maternal self‐report: maternal age (< 20 years; 20 to 34 years; 35 years or older), maternal skin color (White, Black, Mixed), quintiles of family income, years of maternal schooling (0–4, 5–8, 9–11, 12, or older), all collected cross‐sectionally, without recall period. Maternal skin color was self‐reported according to the standard categories used by the Brazilian Institute of Geography and Statistics (IBGE) [18]: White, Black, Brown (Mixed), Yellow, and Indigenous. For the analyses, participants were categorized as White, Black, or Mixed (Brown). Participants classified as Indigenous (*n* = 8) or Yellow (*n* = 15) were excluded due to small numbers. We also included the lack of access to health services, assessed by the question: “Since your child turned 4 years old, has he/she needed a medical appointment, vaccination, and/or hospitalization, but you were unable to access the services?” (yes/no).

The child assessments evaluated biological sex (male, female), cohabitation with an older adult (yes/no), outdoor exposure during the pandemic (almost always/always, never/rarely), and separation from a relative due to COVID‐19 (yes/no). We considered this separation a proxy for close contact with confirmed or suspected cases, including relatives working in healthcare settings. Information was also collected on the child's experience of the death of a close person due to COVID‐19 (yes/no). Information on physician‐diagnosed asthma or bronchitis (yes/no), obesity (defined BMI‐for‐age ≥ +3 z‐scores) (yes/no), and receipt of at least one COVID‐19 vaccine dose (yes/no) was also collected. Data on COVID‐19 vaccination were collected from immunization cards and the National Immunization Program Information System. For cohabitation with an older adult, outdoor exposure, separation from a relative due to COVID‐19, and the death of a close person due to COVID‐19, the recall period covered the entire pandemic until the interview date.

We developed two investigator‐defined scores to characterize patterns of medicine and supplement use. The first, the child COVID‐19 burden score, included negative experiences and COVID‐19 risk factors: (1) Death of a close person or relative due to COVID‐19, (2) separation from a relative due to COVID‐19, (3) cohabitation with an older adult during the pandemic, (4) child has asthma or bronchitis, and (5) child's obesity. The second, the pandemic preventive behavior score, included child COVID‐19 vaccine uptake (at least one dose) and outdoor exposure during the pandemic (never or rarely leaving home). The child COVID‐19 burden score was categorized into 0, 1, 2, or 3 or more factors, and the preventive behavior score was defined as 0, 1, or 2 factors. The scores were weighted equally because no evidence supports differential weighting of their components, and equal weighting avoided assigning arbitrary importance to any single component.

### 2.4 Statistical Analysis

Data were analyzed using STATA 19 (StataCorp., College Station, Texas, USA). We described sample characteristics and the point prevalence (%) of nonevidence‐based medicine and supplement use according to covariates, with respective 95% Confidence Intervals (95% CIs). Point prevalence was also described according to the number of factors in the COVID‐19 burden and preventive behavior scores. The analyses included only children with complete data for the variables comprising each score. We used Pearson's chi‐squared test to assess associations and the chi‐squared test for trend to assess trends across ordinal categorical variables. The number (*N*) and frequency (%) of medicines and supplements were described individually according to therapeutic indication (prophylactic, therapeutic, or both) and physician prescription.

Missing data was handled using complete‐case analysis. Multivariable analyses included participants with complete information on all variables included in the corresponding model. Based on a two‐level conceptual framework [19] (Figure S2), we used hierarchical logistic regression to obtain the Odds Ratios (ORs) and 95% CIs for factors associated with two outcomes in unadjusted and adjusted analyses: (1) off‐label medicine use only (yes/no) and (2) nonevidence‐based supplement use only (yes/no). Two statistical steps were tested according to the conceptual framework based on the existing literature on determinants of medicine use and COVID‐19‐related behaviors. The first included sociodemographic variables (family income, maternal skin color, maternal schooling, maternal age, and child's biological sex). The second included COVID‐19‐related variables (cohabitation with an older adult during the pandemic, separation from a relative due to COVID‐19, death of a close person due to COVID‐19, asthma or bronchitis, obesity, lack of access to health services, and COVID‐19 vaccination). Model building was performed using backward stepwise selection. Statistical significance was defined as *p* < 0.05.

Descriptive analyses were conducted separately for the pre‐ and postvaccine‐availability periods, showing similar distributions of participant characteristics overall, although “separation from a relative” and “the death of a close person due to COVID‐19” differed significantly (*p* = 0.001) (Table S2). Stratified analyses by vaccine availability showed that off‐label medicine use was higher among unvaccinated children before and after vaccine availability (OR_pre_ = 2.20, 95% CI 1.22–3.95 and OR_post_ = 2.57, 95% CI 1.90–3.48, respectively). The association with nonevidence‐based supplement use remained nonsignificant before and after vaccine availability (Table S3).

A sensitivity analysis was also performed to evaluate the COVID‐19 burden score (Table S4), and the direction and magnitude of the associations remained similar to those observed in the primary analysis. Assessment of variance inflation factors (VIFs) indicated no evidence of multicollinearity in the adjusted models (Table S5). Finally, we performed a sensitivity analysis excluding vaccination status from the adjustment set (Table S6). The estimates remained similar for both medicine and supplement use, suggesting that adjustment for vaccination status did not materially influence the results.

## 3 Results

The sample included the 3098 children assessed at 6–7 years who had valid information on medicine and supplement use after COVID‐19 vaccination became available in the municipality. Comparison between the analytical sample and the complete cohort sample at 6–7 years is shown in Table S7. Baseline sociodemographic and health/COVID‐19 characteristics are presented in Table 1, along with the prevalence of nonevidence‐based medicines and supplements. Children were more likely to have mothers aged 20 to 34 (70.7%), with 9 to 11 years of education (33.5%), and white skin color (71%). Children were more likely to be male (50.2%) and to have received at least one COVID‐19 vaccine dose (78.4%). The prevalence of pediatric use of nonevidence‐based medicines and supplements for COVID‐19 treatment or prevention was 13.9% (95% CI 12.7–15.2) with higher frequencies in children of older mothers (16.7%, *p* < 0.001), with white skin color (16.0%, *p* < 0.001), in the highest family income quintile (20.7%, *p* < 0.001) and whose mothers had more years of schooling (19.1%, *p* < 0.001). Use of off‐label medicines and supplements increased with maternal age, family income, and maternal education. Regarding COVID‐19‐related variables, the use of medicines and supplements was higher among children who were separated from a relative due to COVID‐19 (16.8%, *p* = 0.003) and who were not vaccinated against COVID‐19 (19.6%, *p* < 0.001) (Table 1).

**TABLE 1:** Sample description and use of nonevidence‐based medicines and supplements against COVID‐19 during COVID‐19 pandemic in children enrolled in the 6–7 years follow‐up of the Pelotas Birth Cohort Study (*N* = 3098).

	Sample description		Nonevidence‐based medicines and supplements use (%)	95% CI	*p*
	N	%			
Maternal age (*n* = 3097)					0.001^a^
≤ 19	460	14.8	9.17	6.84–12.17	
20–34	2189	70.7	14.40	13.01–15.93	
≥ 35	448	14.5	16.74	13.56–20.50	
Skin color^b^ (*n* = 3072)					< 0.001
White	2181	71.1	16.06	14.60–17.66	
Black	499	16.2	7.44	5.44–10.10	
Mixed	392	12.7	10.20	7.57–13.61	
Family income (quintiles) (*n* = 3057)					< 0.001^a^
1 (poorest)	638	20.9	8.15	6.26–10.54	
2	619	20.3	12.20	9.81–15.01	
3	596	19.5	13.27	10.77–16.24	
4	572	18.7	15.61	12.85–18.83	
5 (richest)	632	20.6	20.76	17.77–24.10	
Maternal schooling (years) (*n* = 3097)					< 0.001^a^
0 to 4	294	9.5	7.14	4.70–10.70	
5 to 8	829	26.8	10.31	8.41–12.58	
9 to 11	1038	33.5	14.10	12.11–16.36	
12+	936	30.2	19.16	16.76–21.81	
Child's biological sex (*n* = 3098)					0.960
Male	1557	50.2	13.92	12.30–15.74	
Female	1541	49.8	14.98	12.34–15.81	
Child lives with an older adult (*n* = 3098)					0.868
No	2687	86.7	14	12.73–15.36	
Yes	411	13.3	13.70	10.68–17.37	
Child's asthma or bronchitis diagnosis (*n* = 3098)					0.807
No	2184	70.5	14.10	12.65–15.60	
Yes	914	29.5	13.72	11.63–16.11	
Child's obesity (*n* = 2791)					0.191
No	2237	80.1	13.23	11.90–14.70	
Yes	554	19.9	15.40	12.60–18.62	
Lack of access to health services after the child is 4 years old (*n* = 3092)					0.330
No	2598	84.0	14.23	12.94–15.63	
Yes	494	16.0	12.60	0.99–1.58	
Separation from a relative due to COVID‐19 (*n* = 3089)					0.003
No	2187	70.8	12.70	11.42–14.22	
Yes	902	29.2	16.85	14.54–19.43	
Death of a close friend/relative due to COVID‐19 (*n* = 3089)					0.144
No	2494	80.7	13.51	12.22–14.91	
Yes	595	19.2	15.82	13.10–19.01	
COVID‐19 children immunization schedule (*n* = 3098)					< 0.001
Nonvaccinated	669	21.6	19.60	16.75–22.80	
At least one dose	2429	78.4	11.70	10.24–13.29	
Total	3098	100	13.9	12.77–15.22	

Abbreviation: CI: 95% confidence interval.

^a^ Linear trend *p*‐value.

^b^ We suppressed the indigenous (*n* = 8) and yellow people (*n* = 15) due to small number of observations.

The use of off‐label medicines only was slightly lower than the nonevidence‐based supplement use (7.9%, [95% CI 7.0–8.9] and 9.8% [95% CI 8.8–10.9], respectively). Ivermectin (5.2%, 95% CI 4.5–6.0) and azithromycin (3.1%, 95% CI 2.5–3.8) were the most frequently used off‐label medicines, while vitamin C (7.7%, 95% CI 6.8–8.7) and vitamin D (5.6%, 95% CI 4.9–6.5) were the most used nonevidence‐based supplements. Nearly 90% of children who used azithromycin reported a physician prescription. Additionally, one‐third of ivermectin use was physician‐prescribed. Regarding supplements, half of the vitamin D use was prescribed by a physician (Table 2).

**TABLE 2:** Descriptive analyses of nonevidence‐based medicines and supplements against COVID‐19 used during the COVID‐19 pandemic according to therapeutic planning (prophylactic, therapeutic, or both) and frequency of medicines and supplements prescribed by a physician. The 6–7‐year follow‐up of the Pelotas Birth Cohort Study (*N* = 3098).

	Medicines and supplements use		Only prophylactic use		Only therapeutic use		Prophylactic and therapeutic use
	Prevalence *N* (%)	Prescribed by a physician *N* (%)	Prevalence *N* (%)	Prescribed by a physician *N* (%)	Prevalence *N* (%)	Prescribed by a physician *N* (%)	Prevalence *N* (%)
Azithromycin^a^	96 (3.1)	85 (88.5)	19 (19.8)	13 (68.4)	70 (72.9)	66 (94.3)	7 (7.3)
Chloroquine	9 (0.3)	8 (89)	3 (33.3)	2 (66.7)	6 (66.7)	6 (100)	—
Ivermectin	162 (5.2)	52 (32.1)	131 (80.9)	30 (22.9)	25 (15.4)	19 (76)	6 (3.7)
Nitazoxanide	35 (1.1)	22 (62.8)	25 (71.4)	13 (52)	7 (20)	6 (85.7)	3 (8.6)
Vitamin C	239 (7.7)	76 (31.8)	199 (83.2)	49 (24.6)	31 (13)	22 (70.1)	9 (3.8)
Vitamin D	174 (5.6)	87 (50)	137 (78.7)	56 (40.9)	27 (15.5)	22 (81.5)	10 (5.8)
Zinc	103 (3.3)	43 (41.7)	77 (74.7)	23 (29.9)	21 (20.4)	15 (71.4)	5 (4.9)
Total^a^	**431 (13.9)**	**180 (41.7)**	**330 (76.6)**	**100 (30.3)**	**86 (19.9)**	**69 (80.2)**	**15 (3.5)**
Medicines only^a^	**245 (7.9)**	**115 (46.9)**	**164 (66.9)**	**44 (26.8)**	**72 (29.4)**	**64 (88.9)**	**9 (3.7)**
Supplements only	**303 (9.8)**	**115 (37.9)**	**254 (83.8)**	**81 (31.9)**	**37 (12.2)**	**25 (67.6)**	**12 (4)**

*Note:* The last three rows, highlighted in bold, refer to the totals for each set of information: Total (medicines + supplements), Total medicines only, and Total supplements only.

Denominator for overall prevalence estimates: *N* = 3098. Percentages in the “Medicines and supplements use” prevalence column were calculated using the total study population as the denominator. Percentages in the “Only prophylactic use,” “Only therapeutic use,” and “Prophylactic and therapeutic use” prevalence columns were calculated using the corresponding total number (*N*) reported in the “Medicines and supplements use” column for each medicine or supplement as the denominator (e.g., *n* = 96 for azithromycin). Percentages in the “Prescribed by a physician” columns were calculated using the corresponding number reported in the adjacent prevalence column as the denominator (e.g., azithromycin: *n* = 19 for prophylactic use and *n* = 70 for therapeutic use).

^a^ One missing piece of information on prescription for azithromycin.

According to the indication of use, 80.9% of ivermectin was used for COVID‐19 prevention, and 22.9% was physician‐prescribed. Vitamin C, D, and zinc were mainly used to prevent COVID‐19 (83.2%, 78.7%, and 74.7%, respectively). Regarding the therapeutic use of off‐label medicines, 72.9% of azithromycin was used for this purpose. Notably, 13% of vitamin C, 15.5% of vitamin D, and 20.4% of zinc were used for COVID‐19 treatment (Table 2).

**FIGURE 1:**
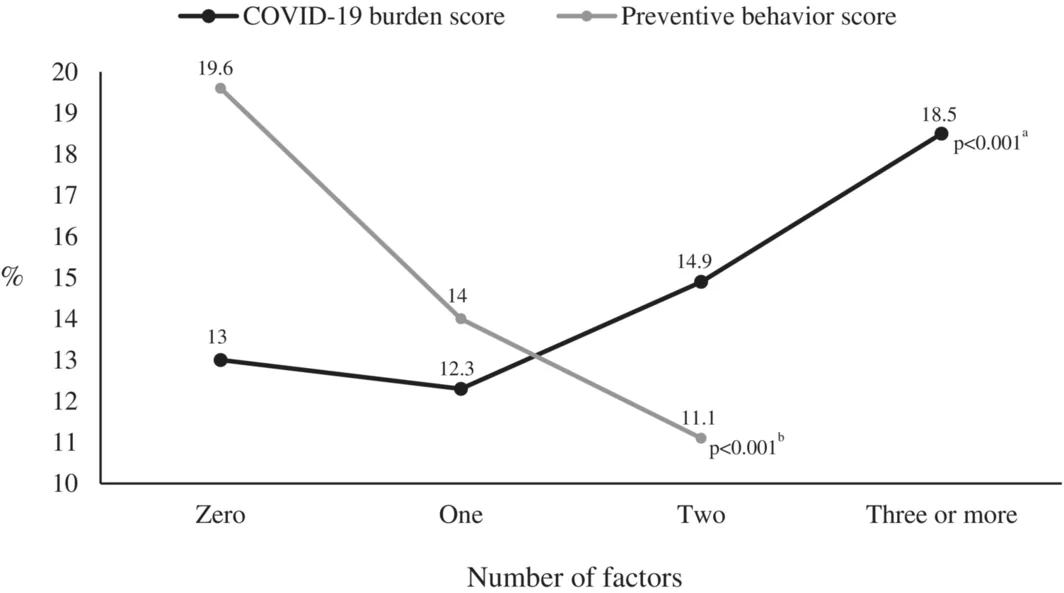
Prevalence (%) of nonevidence‐based medicine and supplement use for prevention or treatment of COVID‐19, according to the number of factors in COVID‐19 burden (*N* = 2789) and preventive behavior (*N* = 3085) scores*. ^a^Pearson's chi‐squared. ^b^Pearson's chi‐squared for trend. *Child's COVID‐19 burden score considered (1) Death of a close person or relative due to COVID‐19, (2) separation from a relative due to COVID‐19, (3) child lives with an older adult, (4) child has asthma or bronchitis, and (5) child's obesity. Child's vaccine uptake and never/rarely outdoor exposure during the pandemic were considered regarding preventive behavior score.

Concerning scores, the use of nonevidence‐based medicines and supplements for prevention or treatment of COVID‐19 increased with the number of factors of the COVID‐19 burden score increased (5.5 percentage points [p.p.] from zero to three or more factors, *p* < 0.001), whereas the use decreased with the preventive behavior score, from 19.6% among children with zero factors to 11.1% among those with two factors (*p* < 0.001) (Figure 1).

In the adjusted analysis, children separated from a relative due to COVID‐19 had a higher chance of using off‐label medicines (OR = 1.37, 95% CI 1.01–1.85). Unvaccinated children had 2.57‐fold higher odds of using off‐label medicines than vaccinated children (Table 3).

**TABLE 3:** Odds ratios of independent variables associated with the use of off‐label medicines against COVID‐19 in children enrolled in the 6–7‐year follow‐up study of the 2015 Pelotas Birth Cohort Study (*n* = 2729).

Variable	Unadjusted			Adjusted^a^		
	OR	95% CI	*p*	OR	95% CI	*p*
Family income (quintiles) (ref: 1st)			< 0.001^b^			0.341
2	1.63	1.00–2.66		1.41	0.83–2.36	
3	1.96	1.21–3.15		1.34	0.78–2.27	
4	2.09	1.30–3.37		1.48	0.86–2.52	
5 (richest)	2.89	1.85–4.53		1.80	1.04–3.13	
Skin color (ref: black)			< 0.001			0.031
White	1.99	1.29–3.08		1.45	0.91–2.28	
Mixed	0.94	0.50–1.77		0.73	0.36–1.47	
Maternal schooling (years) (ref: 0–4 years)			< 0.001^b^			0.151
5 to 8	2.00	0.96–4.12		1.98	0.90–4.31	
9 to 11	2.98	1.48–5.98		2.39	1.10–5.14	
12+	3.71	1.85–7.44		2.39	1.07–5.32	
Maternal age (ref: < 20 years)			0.070			0.788
20 to 34	1.57	1.02–2.41		1.16	0.70–1.90	
≥ 35	1.65	0.98–2.78		1.23	0.67–2.24	
Child's biological sex (ref: male)			0.226			0.238
Female	1.17	0.90–1.52		1.19	0.89–1.58	
Child lives with an older adult (ref: no)			0.925			0.676
Yes	1.01	0.69–1.49		1.11	0.71–1.68	
Separation from a relative due to COVID‐19 (ref: no)			0.024			0.040
Yes	1.37	1.04–1.80		1.37	1.01–1.85	
Death of a close friend/relative due to COVID‐19 (ref: no)			0.711			0.486
Yes	0.93	0.67–1.31		0.87	0.60–1.69	
Child's asthma or bronchitis diagnosis (ref: no)			0.260			0.163
Yes	1.17	0.88–1.55		1.25	0.91–1.69	
Child's obesity (ref: no)			0.460			0.442
Yes	1.13	0.81–1.59		1.15	0.80–1.62	
Lack of access to health services (ref: no)			0.452			0.636
Yes	0.86	0.59–1.25		0.91	0.60–1.36	
COVID‐19 children immunization schedule (ref: at least one dose)			< 0.001			< 0.001
Nonvaccinated	2.58	1.97–3.39		2.57	1.90–3.48	

Abbreviations: 95% CI: 95% confidence interval; OR: odds ratio.

^a^ Adjusted for family income, maternal skin color, maternal age, maternal schooling, child's sex, separation from a relative due to COVID‐19, child experienced death of a close friend or relative due to COVID‐19, child's asthma or bronchitis diagnosis, child's obesity, lack of access to health services and child's COVID‐19 unvaccination.

^b^ Pearson's chi‐squared for trend < 0.001.

In the adjusted analysis of nonevidence‐based supplement use for COVID‐19 (Table 4), we observed a linear trend according to maternal schooling, with ORs ranging from 0.89 at the lowest level of schooling to 1.73 at the highest (*p* < 0.001). Additionally, children with mixed skin color had 2.97‐fold higher odds of using nonevidence‐based supplements compared with black children (*p* = 0.012). Regarding COVID‐19 variables, children separated from a relative and those who experienced the death of a close person due to COVID‐19 had 1.32‐fold (95% CI 1.01–1.74) and 1.46‐fold higher odds (95% CI 1.08–1.98) of using nonevidence‐based supplements compared with those who did not experience these events, respectively.

**TABLE 4:** Odds ratios of independent variables associated with the use of nonevidence‐based supplements against COVID‐19 in children enrolled in the 6–7‐year follow‐up study of the 2015 Pelotas Birth Cohort Study (*n* = 2728).

Variable	Unadjusted			Adjusted^a^		
	OR	95% CI	*p*	OR	95% CI	*p*
Family income (quintiles)(ref: 1st)			< 0.001^b^			0.326
2	1.64	1.04–2.56		1.48	0.91–2.39	
3	1.74	1.11–2.71		1.35	0.82–2.21	
4	2.37	1.54–3.63		1.63	1.00–2.65	
5 (richest)	3.07	2.03–4.62		1.63	0.98–2.70	
Skin color (ref: black)			< 0.001			0.012
White	1.63	0.93–2.84		1.76	0.97–3.20	
Mixed	2.5	1.62–3.85		2.97	1.26–3.21	
Maternal schooling (years) (ref: 0–4 years)			< 0.001^b^			0.010^b^
5 to 8	1.04	0.60–1.77		0.89	0.48–1.63	
9 to 11	1.41	0.84–2.35		1.08	0.60–1.91	
12+	2.49	1.51–4.09		1.73	0.94–3.15	
Maternal age (ref: ≤ 19)			< 0.001			0.094
20–34	1.98	1.29–3.03		1.64	0.97–2.75	
≥ 35	2.37	1.44–3.88		1.93	1.06–3.49	
Child's biological sex (ref: male)			0.826			0.766
Female	0.97	0.76–1.23		0.96	0.74–1.24	
Child lives with an older adult (ref: no)			0.701			0.521
Yes	0.93	0.65–1.33		1.13	0.76–1.68	
Separation from a relative due to COVID‐19 (ref: no)			0.003			0.042
Yes	1.45	1.13–1.86		1.32	1.01–1.74	
Death of a close person or relative due to COVID‐19 (ref: no)			0.008			0.012
Yes	1.46	1.11–1.93		1.46	1.08–1.98	
Child's asthma or bronchitis diagnosis (ref: no)			0.853			0.503
Yes	0.97	0.75–1.26		1.1	0.82–1.47	
Child's obesity (ref: no)			0.477			0.838
Yes	1.11	0.82–1.52		0.96	0.70–1.33	
Lack of access to health services (ref: no)			0.211			0.938
Yes	0.8	0.57–1.13		0.98	0.68–1.42	
COVID‐19 children immunization schedule (ref: at least one dose)			0.044			0.309
Nonvaccinated	1.32	1.01–1.74		1.17	0.86–1.60	

Abbreviations: 95% CI: 95% confidence interval; OR: odds ratio.

^a^ Adjusted for family income, maternal skin color, maternal age, maternal schooling, child's sex, separation from a relative due to COVID‐19, child experienced death of a close friend or relative due to COVID‐19, child's asthma or bronchitis diagnosis, child's obesity, lack of access to health services and child's COVID‐19 unvaccination.

^b^ Pearson's chi‐squared for trend < 0.001.

## 4 Discussion

This study described pediatric use of nonevidence‐based medicines and supplements for COVID‐19 treatment and prevention during the pandemic, using data from the 2015 Pelotas Birth Cohort. Ivermectin and azithromycin were the most frequently used off‐label medicines, while vitamins C and D were the most common nonevidence‐based supplements. Nearly 90% of azithromycin use was prescribed, but only one‐third of ivermectin use had a medical prescription. Despite their widespread use, none of these medicines have demonstrated efficacy against COVID‐19, and they may pose substantial health risks [4].

Off‐label medicines are widely used in pediatrics [20, 21] due to limited evidence‐based data, requiring attention from health professionals and regulators [22]. During the COVID‐19 pandemic in Brazil, misinformation contributed to inappropriate medicines use [4]. The use of nonevidence‐based medicines and supplements in our study was higher among children of older, white, wealthier, and more educated mothers, as well as among those exposed to adverse pandemic experiences (separation from a relative due to COVID‐19 and unvaccinated children).

These patterns may reflect a combination of factors, including greater exposure to misinformation, easier access to healthcare services, higher purchasing power to acquire medicines and supplements, more frequent healthcare encounters, heightened parental anxiety among higher socioeconomic groups, and greater engagement with health‐related information, including social media [14, 15]. Notably, a nationwide Brazilian study conducted during the COVID‐19 pandemic showed that the proportion of adults who failed to seek care despite being ill since the pandemic began was lower in the wealthiest quintile than in the poorest quintile (8.6% vs. 14.1%, respectively) [7], suggesting greater access to healthcare among higher socioeconomic groups. Another possible explanation is that this socioeconomic profile may also reflect the political context in Brazil during the COVID‐19 pandemic. Electoral studies have shown that the political movement aligned with the federal government was initially more prevalent among individuals with higher income and educational attainment, particularly during the 2018 presidential election [23]. During the pandemic, this political alignment was associated with lower adherence to evidence‐based public health measures and greater susceptibility to misinformation [24, 25], which may have contributed to nonevidence‐based medicine and supplement use. Additionally, sales of the “COVID kit” increased substantially in Brazil during the pandemic [8], even though these medicines and supplements were promoted in the Public Health System with no costs [4]. Because some of these factors were not directly measured, our findings should be interpreted as potential explanations rather than causal mechanisms.

Families with negative experiences, comorbidities, or older cohabitants were more likely to adopt off‐label treatments. Conversely, adherence to WHO recommendations (vaccination and reduced outdoor exposure) was correlated with lower use. Sensitivity analyses supported the robustness of the COVID‐19 burden score and highlighted the central contribution of its social components, as excluding these variables attenuated the observed associations (Table S4).

Indeed, Brazilian families with children (0–9 years) were more likely to comply with social distancing and remain at home most of the time during COVID‐19 than families with adolescents and adults [26].

Also, we observed that off‐label medicine use was more frequent among unvaccinated children (the use was 2.57 times higher than among those who received at least one COVID‐19 vaccine dose). It is possible to consider that families skeptical of vaccination may have been more likely to use ivermectin or other unproven therapies. A study using data from the 2015 Pelotas Birth Cohort showed that children who did not initiate the two‐dose primary COVID‐19 vaccine series were more likely to use nonrecommended medicines or vitamins for COVID‐19 [27]. In contrast, nonevidence‐based supplements were associated with mixed skin color and higher levels of maternal schooling and were independent of immunization. A study using data collected from mothers in the same cohort found similar patterns of vitamin use, which were more frequent among white and higher‐income strata [28].

During the COVID‐19 pandemic, disinformation undermined adherence to essential measures in Brazil, fueling distrust in vaccines and promoting ineffective therapies that provided a false sense of security [1]. In 2020, the Federal Board of Medicine and the Ministry of Health authorized and guided the off‐label use of hydroxychloroquine and azithromycin [4]. Medicines such as azithromycin, hydroxychloroquine, ivermectin, and nitazoxanide were prescribed in Brazil even for unconfirmed cases, as part of the so‐called “COVID kit” promoted by medical organizations [4] advocating early treatment and nonmandatory vaccination. The off‐label use of these medicines increased despite uncertainty about their risks to patients with COVID‐19 [29].

The finding that almost 90% of azithromycin use was physician‐prescribed, including 68% of prophylactic use, suggests that inappropriate prescribing practices may have contributed substantially to pediatric exposure. Although antibiotics should be reserved for suspected bacterial infections, the prominence of respiratory symptoms and the uncertainty surrounding COVID‐19 management may have encouraged unnecessary prescribing [30]. These findings highlight the potential contribution of healthcare professionals to inappropriate azithromycin use among children during the pandemic.

Azithromycin is a broad‐spectrum antibiotic, and its indiscriminate use can lead to bacterial resistance and cardiac risks, particularly when combined with chloroquine or hydroxychloroquine. A study by Gérard et al. [31] showed severe adverse cardiac reactions associated with chloroquine and azithromycin use, used either alone or in combination, for the treatment of COVID‐19. A meta‐analysis showed that azithromycin was the most used antibacterial for treating COVID‐19 in children under 13 years old [13]. The authors highlighted the controversial use of azithromycin, ivermectin, and hydroxychloroquine at the time, despite their disapproval by health authorities [13]. The inappropriate use of ivermectin, an antiparasitic drug with multiple uses in humans, can lead to toxicity [32, 33], including severe neurological effects [34].

In our study, few children used chloroquine or hydroxychloroquine, and most uses were physician‐prescribed, but both drugs cause cardiac toxicity and lack efficacy for COVID‐19. Chloroquine and hydroxychloroquine are antimalarial and antirheumatic drugs, whose use was expanded for COVID‐19 treatment, primarily after Yao et al. [35] demonstrated in vitro antiviral activity against SARS‐CoV‐2. The inappropriate use of chloroquine and hydroxychloroquine can lead to severe and irreversible cardiac damage, even at therapeutic doses [36, 37, 38, 39]. Additionally, these medicines cause direct myocardial toxicity and exacerbate underlying myocardial dysfunction [37]. Importantly, there was a lack of chloroquine for individuals with autoimmune diseases, such as rheumatoid arthritis and lupus, who experienced difficulties accessing the medicine during the pandemic [40].

Vitamins and zinc are likely to be used for nonevidence‐based purposes since have not demonstrated efficacy against COVID‐19 [13]. Despite the absence of substantial evidence recommending their use against COVID‐19 [41, 42], global sales of supplements increased by 50% between 2018 and 2020 [43]. The high prevalence of vitamin C, D, and zinc use for prevention (> 75%) observed in our study may be attributed to their affordability and the strong influence of social media on these supplements during the pandemic [44]. The COVID‐19 pandemic triggered self‐protective behaviors, including an increase in purchase intention for dietary supplements [45]. For instance, vitamin D was influenced by the infodemic of misinformation and disinformation, often found in food science and nutrition [46]. Given this context, individuals with a false sense of protection may underestimate risks during COVID‐19 and show low adherence to preventive behaviors [47].

The 2015 Pelotas Birth Cohort is a large population‐based cohort with high response rates and methodological robustness. Its population‐based design and high follow‐up rate support the external validity of the findings for similar urban settings in Brazil. The follow‐up conducted from November 2021 to December 2022 captured medicine use related to the claimed “COVID Kit” and the “Early Treatment of COVID‐19” [4].

Our findings have some limitations. First, because the data were collected through maternal self‐report, recall bias may have occurred. Recall accuracy may have differed according to the child's COVID‐19 status. Mothers of children who had COVID‐19 may have recalled off‐label medicine use more accurately, whereas mothers of children without COVID‐19 may have better recalled preventive use. Similarly, highly publicized drugs such as ivermectin may be recalled more accurately than other therapies. Second, the time between the nonevidence‐based use of medicines and supplements and the interview may have led to recall error, underestimating the prevalence of use, since the recall period covered the entire pandemic. Nevertheless, the unprecedented nature and widespread public impact of the pandemic may have reduced recall error compared with routine circumstances. Recall accuracy may also have varied according to families' engagement with COVID‐19‐related information, potentially resulting in differential misclassification. Third, recall bias may also have affected independent variables (e.g., asthma or bronchitis), given the recall period starting when the child was 4 years old, potentially underestimating the associations. The variable measuring outdoor exposure during COVID‐19 may not fully reflect preventive behavior, because families who had to leave home during the pandemic (e.g., to practice social distancing from relatives) could have had greater outdoor exposure, although this behavior may itself represent a preventive measure. Finally, the temporal ordering between vaccination status and medicine and supplement use could not be established and should therefore be interpreted with caution.

We found that off‐label COVID‐19 medicine use was associated with children's skin color, separation from a relative due to COVID‐19, and lack of COVID‐19 vaccination, whereas nonevidence‐based supplement use was associated with maternal schooling, children's skin color, separation from a relative due to COVID‐19, and the death of a close person due to COVID‐19. Ivermectin and azithromycin were the most frequently used off‐label medicines, while vitamin C and vitamin D were the most frequently used supplements. The COVID‐19 burden and preventive behavior scores may serve as proxies for health‐related behaviors during health emergencies. Further population‐based studies are needed to understand pediatric medicine‐use patterns.

## Author Contributions

**Andréa Dâmaso Bertoldi:** funding acquisition, conceptualization, methodology, data collection, writing, review and editing. **Fernando Silva Guimarães:** conceptualization, methodology, data collection, writing, review and editing. **Alexandra Crispim Boing:** writing, review and editing. **Marysabel Pinto Telis Silveira:** writing, review and editing. **Pedro Curi Hallal:** funding acquisition, writing, review and editing. **Mariângela Freitas da Silveira:** funding acquisition, writing, review and editing.

## Funding

Wellcome Trust, Grant number: 095582/Z/11/Z and 210735/Z/19/Z; Coordination for the Improvement of Higher Education Personnel (CAPES), Grant number: 2207/2012. Mariangela Freitas da Silveira and Andréa Dâmaso Bertoldi are researcher productivity grant holders' level 1D of the Conselho Nacional de Desenvolvimento Científico e Tecnológico [CNPq (National Council for Scientific and Technological Development)]. Fernando Silva Guimarães is in a post doc position funded by Umane and the Department of Science and Technology (DECIT) from the Brazilian Ministry of Health.

## Ethics Statement

The authors declare no competing interests. All involved persons gave their written informed consent prior to study inclusion. The study was approved by the Ethical Committee at the Federal University of Pelotas (UFPel) (CAAE: 51789921.1.0000.5317).

## Conflicts of Interest

The authors declare no conflicts of interest.

## Supporting information


**Figure S1:** Survey window timeline and analytic sample flowchart. The 2015 Pelotas Birth Cohort study.
**Table S1:** Questionnaire on nonevidence‐based medicines and supplements used in the 6–7‐year follow‐up. The 2015 Pelotas Birth Cohort study.
**Figure S2:** Conceptual framework of pediatric use of off‐label medicines and supplements for COVID‐19 treatment or prevention.
**Table S2:** Comparison between samples pre‐ and postvaccine availability for COVID‐19 in children enrolled in the 6–7‐year follow‐up study of the 2015 Pelotas Birth Cohort Study.
**Table S3:** Odds Ratios of independent variables associated with the use of off‐label medicines and nonevidence‐based supplements against COVID‐19 in children enrolled in the 6–7‐year follow‐up study of the 2015 Pelotas Birth Cohort Study, according to pre (*N* = 733) and post (*N* = 2729) vaccine availability.
**Table S4:** Sensitivity analysis of the COVID‐19 burden score. The 2015 Pelotas Birth Cohort.
**Table S5:** Variance Inflation Factors for all covariates included in regression models.
**Table S6:** Adjusted Logistic Regression analysis on off‐label medicine and nonevidence‐based supplement use, excluding vaccination status from the adjustment. The 2015 Pelotas Birth Cohort study.
**Table S7:** Comparison between the complete 6–7 years sample and the analytical sample. Pelotas Birth Cohort study, 2015.

## Data Availability

The data that support the findings of this study are available on request from the corresponding author. The data are not publicly available due to privacy or ethical restrictions.
