# Supplementary material for: Pediatric Use of Off‐Label Medicines and Nonevidence‐Based Supplements for COVID‐19: Findings From the 2015 Pelotas Birth Cohort Study (Brazil)
Source: Pharmacoepidemiol Drug Saf. 2026 Aug 23;35(9):e70460. doi: 10.1002/pds.70460 (PMC13500738; doi:10.1002/pds.70460)
Supplement: Supplementary file 1 [file PDS-35-e70460-s001.docx]

**Supplementary Figure 1.** Survey window timeline and analytic sample flowchart. The 2015 Pelotas Birth Cohort study.

| **Supplementary Table 1.** Questionnaire on non-evidence-based medicines and supplements used in the 6-7-year follow-up. The 2015 Pelotas Birth Cohort study. | |
| --- | --- |
| **COVID-19 variables** | |
| Did <Child> use any of these medicines during the pandemic to prevent or treat COVID-19? | Azithromycin (yes/no)  Chloroquine/hydroxychloroquine (yes/no)  Ivermectin (yes/no)  Nitazoxanide (yes/no)  Vitamin C (yes/no)  Vitamin D (yes/no)  Zinc (yes/no) |
| For each “yes”, please ask: |  |
| Was it prescribed by a doctor? | 0 No 1 Yes 9 IGN |
| Was it used after the diagnosis was confirmed by a laboratory test? | 0 No 1 Yes 9 IGN |
| Was it used for prevention or treatment? | 0 Prevention 1 Treatment 2 Both |

**Supplementary Figure 2.** Conceptual framework of pediatric use of off-label medicines and supplements for COVID-19 treatment or prevention.

| **Supplementary Table 2.** Comparison between samples pre and post vaccine availability for COVID-19 in children enrolled in the 6–7-year follow-up study of the 2015 Pelotas Birth Cohort Study. | | | | |
| --- | --- | --- | --- | --- |
| **Variables** | Pre (n=769) |  | Post (n=3,098) | p-value |
|  | N(%) |  | N(%) |  |
| **Family income (quintiles)** |  |  |  | 0.099 |
| 1 (poorest) | 155(20.3) |  | 638(20.8) |  |
| 2 | 165(21.6) |  | 619(20.3) |  |
| 3 | 157(20.5) |  | 596(19.5) |  |
| 4 | 160(20.9) |  | 572(18.7) |  |
| 5 (richest) | 127(16.7) |  | 632(20.7) |  |
| **Skin color^a^** |  |  |  | 0.944 |
| White | 542(71) |  | 2,181(71) |  |
| Black | 123(16) |  | 499(16) |  |
| Mixed | 101(13) |  | 392(13) |  |
| **Maternal schooling (years)** |  |  |  | 0.002 |
| 0 to 4 | 51(7) |  | 294(9) |  |
| 5 to 8 | 178(23) |  | 829(27) |  |
| 9 to 11 | 304(39) |  | 1,038(33.5) |  |
| 12+ | 236(31) |  | 936(30.3) |  |
| **Maternal age** |  |  |  | 0.965 |
| <20 | 111(14.4) |  | 460(14.8) |  |
| 20 to 34 | 546(71) |  | 2,189(70.7) |  |
| ≥35 | 112(14.6) |  | 448(14.5) |  |
| **Child’s biological sex** |  |  |  | 0.283 |
| Male | 396(51.5) |  | 1,557(50.2) |  |
| Female | 373(48.5) |  | 1,541(49.8) |  |
| **Child lives with an older adult** |  |  |  | 0.305 |
| Yes | 96(13) |  | 411(14) |  |
| **Separation from a relative due to COVID-19** |  |  |  | 0.001 |
| Yes | 175(23) |  | 902(29) |  |
| **Death of a close friend/relative due to COVID-19** |  |  |  | 0.001 |
| Yes | 198(26) |  | 595(19.3) |  |
| **Child’s asthma or bronchitis diagnosis** |  |  |  | 0.097 |
| Yes | 246(32) |  | 914(29.5) |  |
| **Child’s obesity** |  |  |  | 0.001 |
| Yes | 194(26.3) |  | 554(20) |  |
| **Lack of access to health services** |  |  |  | 0.006 |
| Yes | 95(13) |  | 494(16) |  |
| **COVID-19 children immunization schedule** |  |  |  | 0.004 |
| Non-vaccinated | 133(17) |  | 669(21) |  |
| **Off-label use of medicines** |  |  |  | 0.260 |
| Yes | 67(8.7) |  | 245(7.9) |  |
| **Non-evidence-based use of supplements** |  |  |  | 0.085 |
| Yes | 89(11.5) |  | 303(9.8) |  |
| **Non-evidence-based use of medicines and supplements** |  |  |  | 0.099 |
| Yes | 122(15.8) |  | 431(13.9) |  |
| ^a^We suppressed the indigenous (n=8) and yellow people (n=15) due to small number of observations. | | | | |

| **Supplementary Table 3.** Odds Ratios of independent variables associated with the use of off-label medicines and non-evidence-based supplements against COVID-19 in children enrolled in the 6–7-year follow-up study of the 2015 Pelotas Birth Cohort Study, according to pre (N=733) and post (N=2,729) vaccine availability. | | | | | |
| --- | --- | --- | --- | --- | --- |
|  | Use of off-label medicines | | | | |
| **Variable** | Pre vaccine | |  | Post Vaccine | |
|  | OR | p-value |  | OR | p-value |
| **COVID-19 children immunization schedule (ref: at least one dose)** |  | 0.001 |  |  | 0.001 |
| Non-vaccinated | 2.20 |  |  | 2.57 |  |
|  | Use of non-evidence-based supplements | | | | |
| **Variable** | Pre vaccine | |  | Post vaccine | |
|  | OR | p-value |  | OR | p-value |
| **COVID-19 children immunization schedule (ref: at least one dose)** |  | 0.17 |  |  | 0.29 |
| Non-vaccinated | 1.51 |  |  | 1.17 |  |
| OR: Odds ratio | | | | | |

| **Supplementary Table 4.** Sensitivity analysis of the COVID-19 burden score. The 2015 Pelotas Birth Cohort. | | | | | | |
| --- | --- | --- | --- | --- | --- | --- |
| COVID-19 burden score | Zero | One | Two | Three or more | p-value | N |
| Paper score | 13 | 13.1 | 15.1 | 19.4 | 0.01 | 3,529 |
| A | 13.6 | 12.9 | 16.5 | 18.1 | 0.06 | 3,529 |
| B | 13.0 | 14.1 | 16.3 | 14.7 | 0.2 | 3,529 |
| C | 12.5 | 13.7 | 15.8 | 19.8 | 0.02 | 3,529 |
| D | 12.2 | 14.5 | 16.0 | 21.5 | <0.01 | 3,529 |
| E | 13.7 | 13.1 | 17.2 | 18.4 | 0.02 | 3,857 |
| F | 13.2 | 14.5 | 17.8 | 25.0 | 0.02 | 3,857 |
| Composite scores: (A) exclusion of death of a close person or relative due to COVID-19, (B) exclusion of separation from a relative due to COVID-19, (C) exclusion of child lives with an old person, (D) exclusion of child has asthma or bronchitis, (E) exclusion of child’s obesity and (F) exclusion of comorbidities (asthma or bronchitis and obesity). | | | | | | |

| **Supplementary Table 5.** Variance Inflation Factors for all covariates included in regression models. | |
| --- | --- |
| **Variable** | **Variance Inflation Factor (VIF)** |
| Biological sex | 1.02 |
| Family income |  |
| 2 | 1.59 |
| 3 | 1.66 |
| 4 | 1.78 |
| 5 (richest) | 2.22 |
| Skin color |  |
| White | 1.55 |
| Mixed | 1.63 |
| Maternal age |  |
| 20 to 34 | 1.94 |
| ≥35 | 1.94 |
| Maternal schooling |  |
| 5 to 8 | 2.86 |
| 9 to 11 | 3.31 |
| 12+ | 3.75 |
| Child lives with an older adult | 1.06 |
| Death of a close friend/relative due to COVID-19 | 1.01 |
| Child's asthma or bronchitis | 1.03 |
| Child's obesity | 1.02 |
| Lack of access to health services | 1.03 |
| Separation from a relative due to COVID-19 | 1.03 |
| COVID-19 children's immunization status | 1.01 |
| **Mean VIF** | 1.71 |

| **Supplementary Table 6.** Adjusted Logistic Regression analysis on off-label medicine and non-evidence-based supplement use, excluding vaccination status from the adjustment. The 2015 Pelotas Birth Cohort study. | | | | | | | | | | | |
| --- | --- | --- | --- | --- | --- | --- | --- | --- | --- | --- | --- |
|  | Off-label medicine use | | | | |  | Non-evidence-based supplement use | | | | |
| Variables | 1 | |  | 2 | |  | 3 | |  | 4 | |
|  | OR | 95% CI |  | OR | 95% CI |  | OR | 95% CI |  | OR | 95% CI |
| **Family income (quintiles) (ref: 1^st^)** |  |  |  |  |  |  |  |  |  |  |  |
| 2 | 1.4 | 0.8-2.4 |  | 1.4 | 0.9-2.4 |  | 1.5 | 0.9-2.3 |  | 1.5 | 0.9-2.4 |
| 3 | 1.3 | 0.8-2.3 |  | 1.4 | 0.8-2.3 |  | 1.3 | 0.8-2.2 |  | 1.4 | 0.8-2.2 |
| 4 | 1.5 | 0.9-2.5 |  | 1.6 | 0.9-2.7 |  | 1.6 | 1.0-2.6 |  | 1.6 | 1.0-2.6 |
| 5 (richest) | 1.8 | 1.0-3.1 |  | 1.9 | 1.1-3.3 |  | 1.6 | 1.0-2.7 |  | 1.6 | 1.0-2.7 |
| **Skin color^a^ (ref: black)** |  |  |  |  |  |  |  |  |  |  |  |
| White | 0.7 | 0.4-1.5 |  | 0.7 | 0.4-1.4 |  | 1.8 | 1.0-3.2 |  | 1.8 | 1.0-3.1 |
| Mixed | 1.4 | 0.9-2.3 |  | 1.5 | 1.0-2.4 |  | 2.0 | 1.2-3.2 |  | 2.0 | 1.2-3.2 |
| **Maternal schooling (years) (ref: 0 to 4 years)** |  |  |  |  |  |  |  |  |  |  |  |
| 5 to 8 | 2.0 | 0.9-4.3 |  | 1.8 | 0.8-4.0 |  | 0.9 | 0.5-1.6 |  | 0.9 | 0.5-1.6 |
| 9 to 11 | 2.4 | 1.1-5.1 |  | 2.2 | 1.0-4.6 |  | 1.1 | 0.6-1.9 |  | 1.1 | 0.6-1.9 |
| 12+ | 2.4 | 1.1-5.3 |  | 2.2 | 1.0-5.0 |  | 1.7 | 0.9-3.1 |  | 1.7 | 0.9-3.1 |
| **Maternal age (ref: <20 years)** |  |  |  |  |  |  |  |  |  |  |  |
| 20 to 34 | 1.2 | 0.7-1.9 |  | 1.1 | 0.7-1.8 |  | 1.6 | 1.0-2.7 |  | 1.6 | 1.0-2.7 |
| ≥35 | 1.2 | 0.7-2.2 |  | 1.2 | 0.6-2.1 |  | 1.9 | 1.0-3.4 |  | 1.9 | 1.0-3.4 |
| **Child’s biological sex (ref: male)** |  |  |  |  |  |  |  |  |  |  |  |
| Female | 1.2 | 0.9-1.6 |  | 1.2 | 0.9-1.6 |  | 1.0 | 0.7-1.2 |  | 1.0 | 0.7-1.2 |
| **Child lives with an older adult (ref: no)** |  |  |  |  |  |  |  |  |  |  |  |
| Yes | 1.1 | 0.7-1.7 |  | 1.0 | 0.7-1.6 |  | 1.1 | 0.7-1.6 |  | 1.1 | 0.7-1.6 |
| **Separation from a relative due to COVID-19 (ref: no)** |  |  |  |  |  |  |  |  |  |  |  |
| Yes | 1.4 | 1.0-1.9 |  | 1.3 | 1.0-1.8 |  | 1.3 | 1.0-1.7 |  | 1.3 | 1.0-1.7 |
| **Death of a close friend/relative due to COVID-19 (ref: no)** |  |  |  |  |  |  |  |  |  |  |  |
| Yes | 0.9 | 0.6-1.3 |  | 0.9 | 0.6-1.3 |  | 1.5 | 1.0-1.9 |  | 1.5 | 1.0-1.9 |
| **Child’s asthma or bronchitis diagnosis (ref: no)** |  |  |  |  |  |  |  |  |  |  |  |
| Yes | 1.2 | 0.9-1.7 |  | 1.3 | 0.9-1.7 |  | 1.1 | 0.8-1.4 |  | 1.1 | 0.8-1.5 |
| **Child’s obesity (ref: no)** |  |  |  |  |  |  |  |  |  |  |  |
| Yes | 1.1 | 0.8-1.6 |  | 1.1 | 0.8-1.6 |  | 1.0 | 0.7-1.3 |  | 1.0 | 0.7-1.3 |
| **Lack of access to health services (ref: no)** |  |  |  |  |  |  |  |  |  |  |  |
| Yes | 0.9 | 0.6-1.4 |  | 1.0 | 0.6-1.4 |  | 1.0 | 0.6-1.3 |  | 1.0 | 0.7-1.4 |
| **COVID-19 children immunization schedule (ref: at least one dose)** |  |  |  |  |  |  |  |  |  |  |  |
| Non-vaccinated | 2.6 | 1.9-3.5 |  | - | - |  | 1.2 | 0.9-1.6 |  | - | - |
| OR: Odds Ratio; 95% CI: 95% Confidence Interval.  ^a^We suppressed the indigenous (n=8) and yellow people (n=15) due to small number of observations.  ^1^Adjusted analysis for medicine use, including family income, maternal skin color, maternal age, maternal schooling, child’s sex, separation from a relative due to COVID-19, death of a close friend or relative due to COVID-19, child’s asthma/bronchitis, obesity, lack of access to health services, and child’s COVID-19 unvaccination.  ^2^Same as Model 1, excluding child’s COVID-19 unvaccination.  ^3^Adjusted analysis for supplement use with the same covariates as Model 1.  ^4^ Same as Model 3, excluding child’s COVID-19 unvaccination. | | | | | | | | | | | |

| **Supplementary Table 7.** Comparison between the complete 6-7 years sample and the analytical sample. Pelotas Birth Cohort study, 2015. | | | | | | | |
| --- | --- | --- | --- | --- | --- | --- | --- |
|  | **Analytical sample  (N=3,098)** | | |  | **6-7 years sample  (N=3,867)** | | |
|  | **N** | **%** | **95% CI** |  | **N** | **%** | **95% CI** |
| **Maternal age** |  |  |  |  |  |  |  |
| ≤19 | 460 | 14.8 | 13.6-16.1 |  | 571 | 14.8 | 13.7-15.9 |
| 20-34 | 2,189 | 70.7 | 69.0-72.2 |  | 2,735 | 70.7 | 69.3-72.1 |
| ≥35 | 448 | 14.5 | 13.2-15.7 |  | 560 | 14.5 | 13.4-15.6 |
| **Skin color**^a^ |  |  |  |  |  |  |  |
| White | 2,181 | 71 | 69.3-72.5 |  | 2,723 | 71 | 69.5-72.3 |
| Black | 499 | 16.2 | 15.0-17.6 |  | 622 | 16.2 | 15.0-17.4 |
| Mixed | 392 | 12.7 | 11.6-14.0 |  | 493 | 12.8 | 11.8-13.9 |
| **Family income (quintiles)** |  |  |  |  |  |  |  |
| 1 (poorest) | 638 | 20.9 | 19.4-22.3 |  | 770 | 19.9 | 19.5-22.0 |
| 2 | 619 | 20.3 | 18.9-21.7 |  | 793 | 20.5 | 19.3-21.8 |
| 3 | 596 | 19.5 | 18.2-20.9 |  | 789 | 20.4 | 18.4-20.1 |
| 4 | 572 | 18.7 | 17.3-20.1 |  | 769 | 19.9 | 17.9-20.4 |
| 5 (richest) | 632 | 20.7 | 19.2-22.1 |  | 744 | 19.3 | 18.6-21.1 |
| **Maternal schooling (years)** |  |  |  |  |  |  |  |
| 0 to 4 | 294 | 9.5 | 8.5-10.6 |  | 345 | 8.9 | 8.0-9.8 |
| 5 to 8 | 829 | 26.8 | 23.2-28.3 |  | 1,007 | 26.1 | 24.7-27.4 |
| 9 to 11 | 1,038 | 33.5 | 31.9-35.2 |  | 1,342 | 34.7 | 33.2-36.2 |
| 12+ | 936 | 30.2 | 28.6-31.9 |  | 1,172 | 30.3 | 28.8-31.8 |
| **Children's biological sex** |  |  |  |  |  |  |  |
| Male | 1,557 | 50.2 | 48.5-52.0 |  | 1,953 | 50.5 | 48.9-52.0 |
| Female | 1,541 | 49.8 | 48.0-51.5 |  | 1,914 | 49.5 | 47.9-51.0 |
| **Children live with an older adult** |  |  |  |  |  |  |  |
| No | 2,687 | 86.7 | 85.5-87.9 |  | 3,360 | 86.9 | 85.8-87.9 |
| Yes | 411 | 13.3 | 12.1-14.5 |  | 507 | 13.1 | 12.0-14.2 |
| **Child’s asthma or Bronchitis diagnosis** |  |  |  |  |  |  |  |
| No | 2,184 | 70.5 | 68.8-72.0 |  | 2,707 | 70 | 68.5-71.4 |
| Yes | 914 | 29.5 | 27.9-31.1 |  | 1,160 | 30 | 28.6-31.4 |
| **Child’s obesity** |  |  |  |  |  |  |  |
| No | 2,237 | 80.1 | 78.6-81.5 |  | 2,783 | 78.8 | 77.4-80.1 |
| Yes | 554 | 19.9 | 18.4-21.3 |  | 748 | 21.2 | 19.8-22.5 |
| **Lack of access to health services after the child is 4 years old** |  |  |  |  |  |  |  |
| No | 2,598 | 84 | 82.6-85.2 |  | 3,272 | 84.7 | 83.5-85.8 |
| Yes | 494 | 16 | 14.7-17.3 |  | 589 | 15.3 | 14.1-16.4 |
| **Separation from a relative due to COVID-19** |  |  |  |  |  |  |  |
| No | 2,187 | 70.8 | 69.1-72.3 |  | 2,781 | 72.1 | 70.6-73.4 |
| Yes | 902 | 29.2 | 27.6-30.8 |  | 1,077 | 27.9 | 26.5-29.3 |
| **Death of a close person or relative due to COVID-19** |  |  |  |  |  |  |  |
| No | 2,494 | 80.7 | 79.3-82.0 |  | 3,065 | 79.4 | 78.1-80.7 |
| Yes | 595 | 19.2 | 17.9-20.7 |  | 793 | 20.6 | 19.3-21.8 |
| **Child’s left house during pandemic (outdoor exposure)** |  |  |  |  |  |  |  |
| Almost always/always | 1,926 | 62.4 | 60.7-64.1 |  | 2,402 | 62.3 | 60.7-63.8 |
| Never/almost never | 1,160 | 37.6 | 35.9-39.3 |  | 1,453 | 37.7 | 36.1-39.2 |
| **COVID-19 children immunization schedule** |  |  |  |  |  |  |  |
| Non-vaccinated | 669 | 21.6 | 20.2-23.0 |  | 802 | 20.7 | 19.5-22.0 |
| At least one dose | 2,429 | 78.4 | 76.9-79.8 |  | 3,065 | 79.3 | 77.9-80.5 |
| ^a^We suppressed the indigenous (n=8) and yellow people (n=15) due to small number of observations.  95% CI: 95% Confidence Interval. | | | | |  |  |  |
